# Homeothermy, microclimate and heat tolerance: Evolutionary origins, mechanisms and survival

**DOI:** 10.1007/s00484-026-03272-8

**Published:** 2026-07-07

**Authors:** Frank E. Marino

**Affiliations:** School of Rural Medicine, Charles Sturt University, Orange, NSW 2800 Australia

**Keywords:** Acclimatization, Endothermy, Homeothermy, Thermoregulation, Microclimate, Heat stress, Wet-bulb temperature, Survival

## Abstract

Why mammals defend species-specific body temperatures, often across a range extending from the low 30s to around 40 °C, remains a central question in comparative physiology. In this Commentary, it is suggested that mammalian heat tolerance is best understood not through single environmental thresholds or global mean temperature, but through the interaction of microclimate, heat-balance physics, effector coordination and cardiovascular constraint. Using humans as one focal example within a broader comparative framework, it distinguishes endothermy from homeothermy and then consideration as to how different mammalian lineages solve the problem of heat balance through differing combinations of evaporative cooling, behavioural refuge, water conservation, heterothermy and tolerance of transient body temperature elevation. The discussion then addresses mechanistic pathways of heat exchange, emphasising the importance of radiation, airflow and evaporative opportunity within the immediate microclimate, and present a microclimate-linked Arrhenius/Q_10_ postulate for why a defended body temperature in the high 30 °C range is biologically plausible for some mammalian lineages. The central argument is that humans are not thermoregulatory outliers, but one endothermic solution to environmental heat load, and that the question of future survivability depends on whether specific microclimates remain compensable as radiation, humidity, airflow, metabolic demand, and water availability interact.

## Introduction

Thermal biology links organismal function to environment through the physics of heat exchange and the physiology of control. Mammals and birds are endothermic, producing metabolic heat, and generally homeothermic, maintaining relatively stable body temperatures through coordinated autonomic and behavioral effectors. Humans are thus not exceptional in regulating a core temperature (T_c_) near ~ 37 °C. Rather, we represent one lineage whose thermal ‘operating point’ reflects both deep evolutionary constraints and recent ecological challenges (Crompton et al. 1978; Parsons 2007; Romanovsky 2007; Boyles et al. 2011).

The current climate context has focused interest in “limits” to heat tolerance. Yet everyday narratives often consider risk in single numbers. For instance, global mean surface temperature change, a headline air temperature, or a single wet-bulb threshold (He et al. 2022; Hunt et al. 2023; Bell et al. 2024). For physiology, this might be a category error. Since organisms do not experience global means. Rather, they experience microclimates shaped by latitude, season, time of day, urban geography, solar load, humidity, wind speed, and the thermal properties of surrounding surfaces (Potter et al. 2013; Mitchell et al. 2024). These variables determine radiative and convective heat gain and the feasibility of evaporative heat loss, where evaporation is the dominant pathway for human heat dissipation during substantial heat stress (Lieberman 2015).

**Fig. 1:**
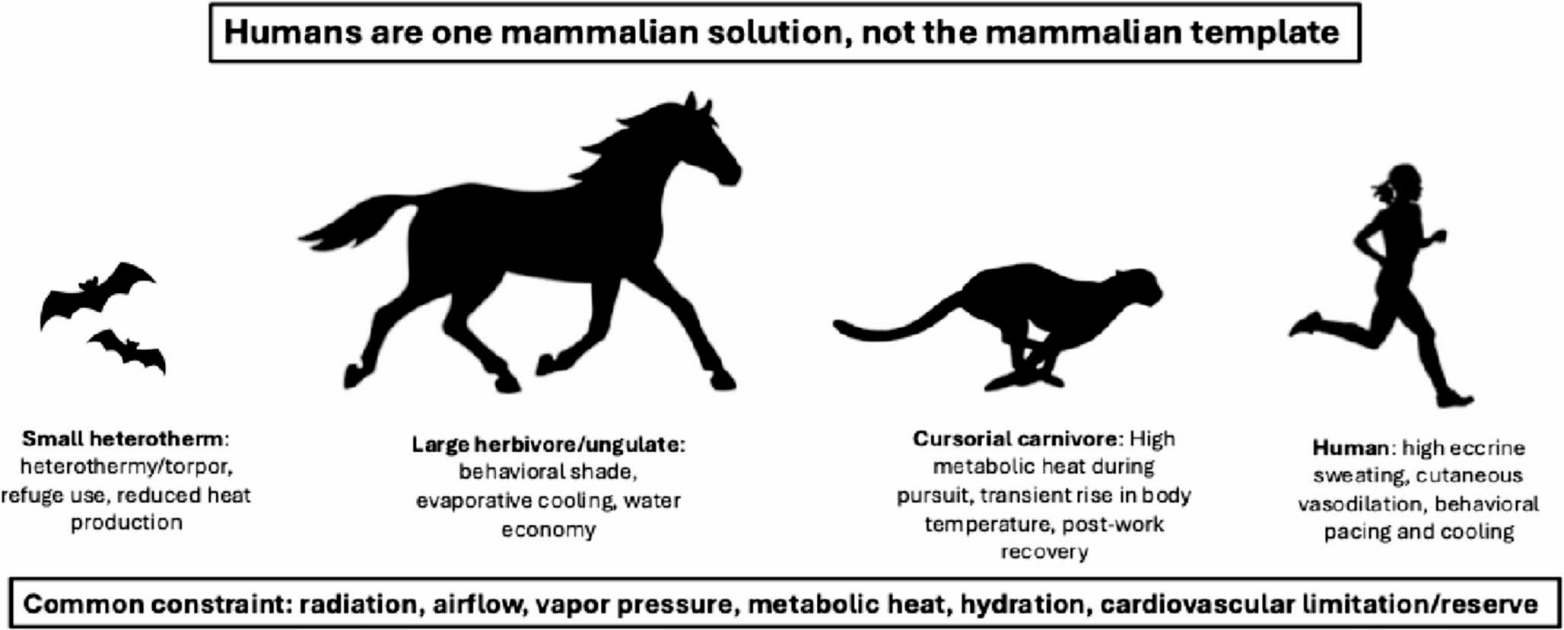
Comparative mammalian solutions to thermal balance. Conceptual schematic illustrating that humans represent one mammalian solution to the problem of heat balance, rather than the mammalian template. Small heterotherms may rely on torpor, refuge and reduced heat production; large herbivores on behavioral shade seeking, evaporative cooling and water economy; cursorial carnivores on tolerating high metabolic heat loads during pursuit with post-exercise recovery; and humans on high eccrine sweating, cutaneous vasodilation, and behavioral pacing and cooling. Across all groups, thermal balance is constrained by the shared influences of radiation, airflow, vapor pressure, metabolic heat production, hydration state and cardiovascular reserve

This commentary re-centers the discussion on (i) comparative biology, where homeothermy is an ancient and flexible strategy rather than a uniquely human one; (ii) control architecture, where multiple effectors with distinct thresholds combine to produce a balance point (Romanovsky 2007; Werner 2010); and (iii) microclimate, where solar radiation and wind are often as important as humidity and air temperature in determining heat strain. To frame this comparison, the major thermoregulatory strategies across taxa, and their possible relevance to performance and climate vulnerability, are summarized in Fig. 1.

The aim is to place human thermoregulation within a broader comparative and mechanistic framework, rather than to treat heat tolerance as a question of single metrics or global mean climate alone. Framed this way, the key question is not whether humans or indeed other mammals can survive climate change in the abstract, but under which combinations of microclimate, activity, water availability and behavioral needs the present thermoregulatory strategy remains compensable and adaptable.

## Definitions and control architecture

Endothermy refers to the predominance of metabolically derived heat in maintaining body temperature (Boyles et al. 2011). Homeothermy refers to the stability of body temperature across time, regardless of whether heat is generated internally or obtained from the environment (Crompton et al. 1978). Behavioral homeothermy can be achieved by ectotherms (e.g., reptiles that bask and shuttle between sun and shade) (Angilette Jr et al. 2002), highlighting that homeothermy is not synonymous with endothermy.

In humans, thermoregulation operates as a distributed control system with multiple sensors (skin, viscera, central nervous system) and multiple effectors (cutaneous vasomotor tone, sweating, shivering, behavioral selection). Importantly, the concept of ‘set point’ can be misleading if it implies a single fixed target. Contemporary models emphasize that effector thresholds can be considered “set points” for each effector (e.g., the T_c_ at which sweating begins), while the overall “balance point” (operating point) emerges from the weighted integration of thermal signals and effector actions (Romanovsky 2007).

**Fig. 2:**
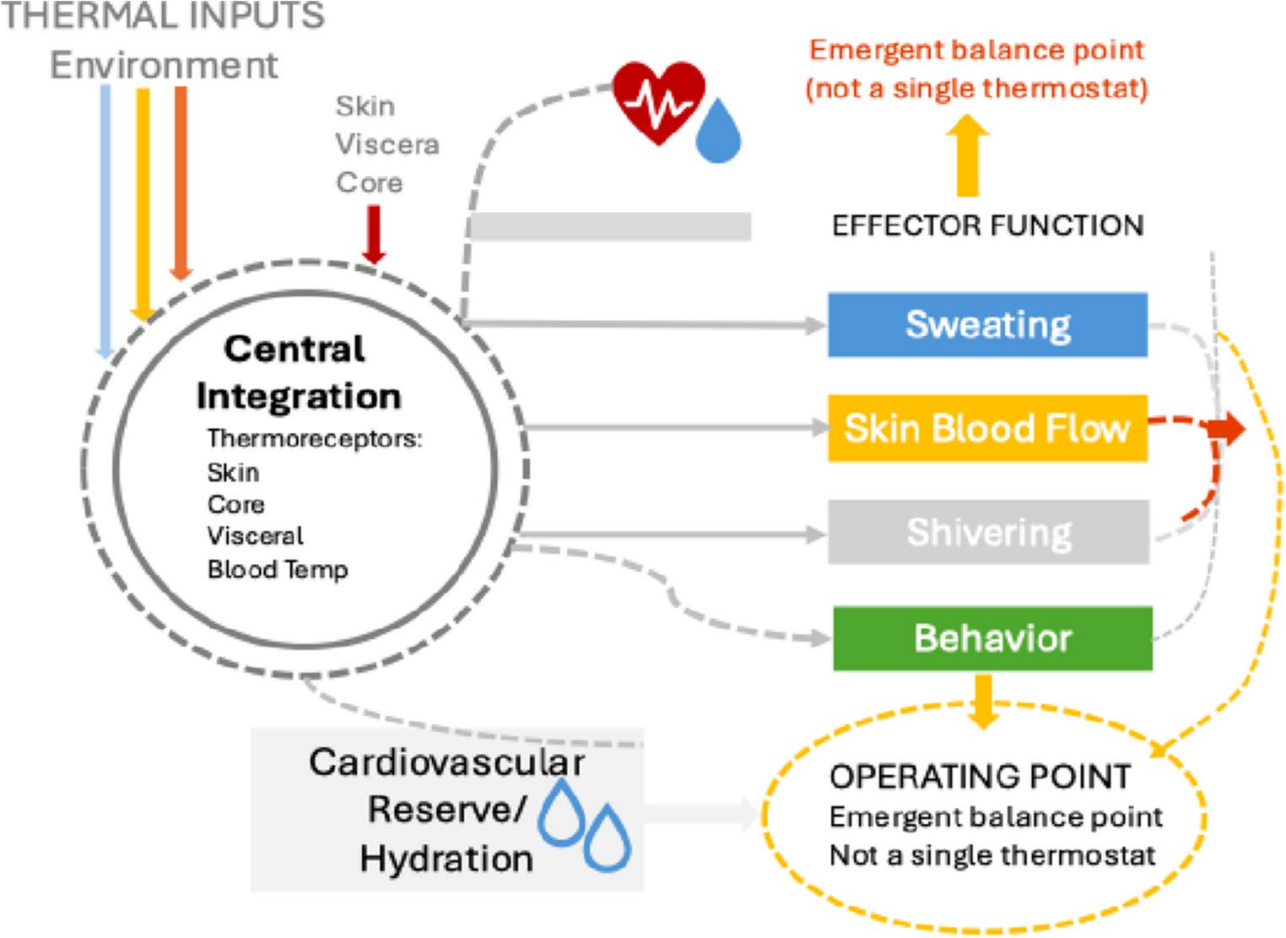
Thermoregulation as an emergent balance point rather than a single thermostat. A conceptual model of thermoregulation in which thermal information from the environment, skin, viscera and core is integrated centrally to influence multiple effectors, including sweating, skin blood flow, shivering and behavior. In this framework, body temperature is not defended by a single thermostat, but reflects an emergent operating point arising from the interaction of afferent inputs, effector capacity, hydration state and available cardiovascular reserve

This framing helps reconcile why regulation can appear stable under some conditions and flexible under others. During mild warming, vasodilation increases dry heat loss, while during more severe warming, sweating dominates (Shido et al. 1999; Cheuvront et al. 2009). During dehydration or cardiovascular strain, effector efficacy changes, whereby sweating may be reduced, or skin blood flow constrained to maintain arterial pressure, shifting the operating point to a higher T_c_. The system is thus adaptive, but not limitless, because effectors are coupled to cardiovascular and fluid-balance constraints (see Fig. 2) (Crandall and González-Alonso 2010; Crandall and Wilson 2015).

Two simple examples illustrate the point. First, a resting person in still, humid heat may sweat profusely but gain little cooling because evaporation is limited by vapor pressure; the effector (sweat) is active, but ineffective (Kerslake 1972; Nielsen 1996; Dennis and Noakes 1999; Marino et al. 2000). Second, during prolonged exercise in the heat, rising heart rate and reduced central blood volume can constrain skin blood flow, limiting heat transfer from core to shell where the effector (vasodilation) is active, but capped by cardiovascular reserve. In both cases, the “limit” is not the inability to generate a response but the inability of the response to achieve the required heat exchange.

### A microclimate-linked, Arrhenius/Q10 heuristic for the ~ 37 °C balance point

A recurring question, especially in climate discussions, is why humans (and many mammals) regulate T_c_ near ~ 37 °C. Rather than treating this as an independent hypothesis as a ‘chicken-and-egg’ problem (did enzymes ‘choose’ 37 °C or did 37 °C ‘choose’ enzymes? ), a complementary, microclimate-linked postulate is more useful. That is, once a lineage commits to endo-homeothermy, the defended T_c_ must sit sufficiently above the typical ambient conditions experienced during its evolutionary history to enable net heat transfer away from the core. In this view, ~ 37 °C can be interpreted as an emergent balance point (Fig. 2) constrained by heat-transfer physics and temperature dependent metabolism, with biochemistry, immunity, and physical performance co-adapting around that operating range.

The key step is an explicit environmental assumption. Homeothermy is only energetically plausible if T_c_ is usually higher than ambient temperature, such that heat can be exported by dry exchange (radiation/convection) when evaporation is limited, and such that the organism can exploit activity across cooler microclimates. Based on long timescale temperature reconstructions and modelling (Haywood et al. 2019), and using a present-day global mean near ~ 10–15 °C as a reference (Rohde et al. 2013), indicates that during parts of the Eocene, temperature anomalies may have been ~ 8–12 °C above late Quaternary baselines (Zachos et al. 2001; Franzen et al. 2009; Tudge 2009). Taken at face value as a broad proxy for the ambient conditions that shaped early primate microclimates (noting the substantial uncertainty and the fact that microclimates differ from global means), this yields an illustrative ambient temperature in the order of ~ 25 °C.

With that ambient temperature anchor, a simple Q_10_-Arrhenius-style argument can be used to relate temperature dependent heat production to the maintenance of a stable T_c_ above the environment. Following the approach used in human heat balance texts (e.g., (Gisolfi et al. 2000), we can assume that the operative core-to-ambient gradient to be: T_c_ − 25 °C). If T_c_ were to rise by 1 °C at the same ambient temperature, the gradient becomes (T_c_ + 1 − 25) = (T_c_ − 24). The relative change in gradient is, therefore, (T_c_ − 24)/(T_c_ − 25). If this is set equal to the approximate per degree metabolic scaling implied by a Q_10_ ≈ 2.3 (i.e., 2.3^0.1^ ≈ 1.086), we obtain:

$$(Tc-24) / (Tc-25) = 1.086$$

**Fig. 3:**
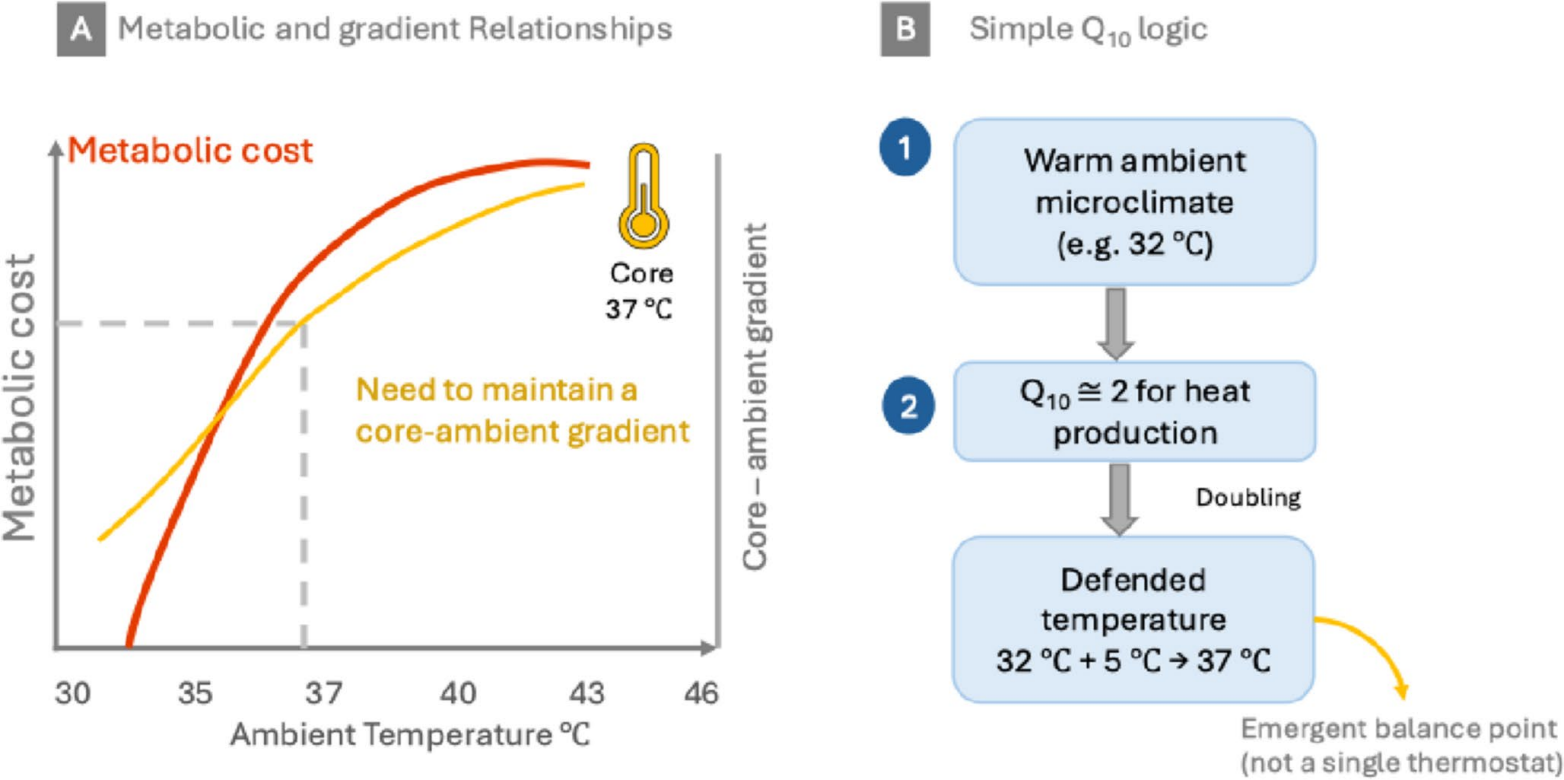
A schematic rationale for why a core temperature near 37°C is plausible. The postulated model shows how a defended body temperature near 37°C may emerge from the interaction between metabolic cost and the need to maintain a core-to-ambient thermal gradient. Panel A illustrates the trade-off between metabolic expenditure and gradient preservation across ambient temperatures. Panel B presents a simplified Q10-based logic in which a warm ambient microclimate may favor an operating point approximately 5°C above ambient, yielding a defended temperature near 37 °C. In this view, 37°C is interpreted as an emergent balance point rather than the product of a single fixed thermostat

Solving yields T_c_ ≈ 36.6 °C, precisely where T_c_ is classically placed as a balance point i.e., ~ 37 °C (Mackowiak et al. 1992; Protsiv et al. 2020). This is not to claim that this calculation ‘explains’ 37°C uniquely, but that it demonstrates plausibility. As illustrated in Fig. 3, a defended T_c_ in the high-30s emerges naturally from (i) a warm ambient assumption and (ii) a temperature-dependent metabolic cost of heat production. Importantly, the argument does not require a specific sequence of causation between enzymes and temperature. The balance point is constrained by the physics and energetics of maintaining the core – ambient gradient where molecular and organ-system function then evolves within those constraints.

There are, of course, limitations. First, ambient temperatures millions of years ago are uncertain, and global means are not equivalent to organismal microclimates. Second, endothermy/homeothermy long predates primate divergences. Thus, the calculation is best interpreted as a postulate for why a balance point is energetically coherent under warm, mid-latitude microclimates rather than as a literal account of the origin of homeothermy. Third, Q_10_ values vary across pathways and temperatures. For these reasons, the model is a plausibility argument, not a derived mechanistic reality.

## Evolutionary context: From early mammals to hominins

Homeothermy and endothermy likely arose early in mammalian evolution and has been repeatedly tuned to the ecological niche. Across mammals, resting T_c_ commonly lies in the mid- to high-30s °C, yet many species demonstrate controlled departures from strict homeothermy. That is, daily heterothermy, torpor and hibernation, when energy balance favors reduced heat production (Ruf and Geiser 2015). This broader comparative view matters because it shows that thermal regulation is a strategy, not a rigid biological mechanism, and that “stable T_c_” does not preclude adaptive variation.

Within hominins, selection pressures in hot, open environments likely elevated the importance of heat dissipation, especially under endurance locomotion (Lieberman 2015). Traits often attributed to *Homo erectus*, elongated lower limbs, relatively reduced body hair, abundant eccrine sweating, and body shapes with higher surface-area-to-mass in hot climates, enhance convection and evaporation. Conversely, Neanderthals are frequently described as having a cold-adapted morphology (more compact builds), a pattern aligned with general ecogeographical rules relating body shape to heat exchange (Tilkens et al. 2007; Marino 2019). The evolutionary point is not that humans are uniquely tolerant of heat, but that human thermoregulation represents one endothermic solution to the problem of sustaining activity in hot environments. Its reliance on evaporation is highly effective when water is available and air movement permits vapor transfer, but that same reliance links heat tolerance to hydration status, humidity, and clothing resistance.

A broader comparative perspective also shows that mammalian thermoregulation is not a single solution, but a spectrum of ecological and physiological strategies shaped by niche. Arid adapted species such as the gerenuk (*Litocranius walleri -* antelope) occupy dry thornbush habitats, feed almost exclusively on shrubs and trees, and appear largely independent of free water, illustrating how morphology, diet and habitat use can reduce thermal and hydric exposure in ways quite different from the human reliance on sweating during sustained activity (Leuthold 1978; Bärmann et al. 2021). In this sense, the human pattern should be regarded not as the default mammalian strategy, but as one endothermic solution among several, each coupling heat balance, water economy and behavior to the demands of a particular ecological setting.

## Mechanistic pathways for heat exchange and physiological coordination

Heat balance can be expressed as *S = M ± R ± C ± K − E*, where *S* is body heat storage, *M* metabolic heat production, *R* radiative exchange, *C* convective exchange, *K* conductive exchange, and *E* evaporative heat loss (Commission 2001). For most real-world heat stress, conduction is minor, while radiative and convective terms depend strongly on solar load, surrounding surface temperatures, posture, clothing, and wind speed.

Radiation is often underestimated in discussions that focus on air temperature alone. Outdoors, direct short-wave solar radiation and reflected radiation from surfaces can impose large heat gains (Martin 1998). Depending on solar intensity, clothing, and exposed surface area, net radiative gain can be comparable to or exceed metabolic heat production during light activity (Otani et al. 2019). Conversely, shade can reduce radiative load dramatically and is often the single most effective behavioral intervention for passive heat exposure (Nielsen et al. 1988). Convection depends on the temperature gradient between the skin - clothing surface and ambient air, and on air movement (Saunders et al. 2005). At air temperatures below skin temperature, wind enhances convective cooling; at air temperatures above skin temperature, wind can increase convective heat gain unless evaporation dominates. In practice, wind often helps because it also increases evaporation by thinning the boundary layer and improving vapor transfer through clothing.

Evaporation becomes the primary avenue for heat dissipation at high heat loads and during exercise (Nielsen 1996). Sweat must evaporate to remove heat; dripping sweat confers little cooling but large fluid loss (Saltin 1964). Evaporative capacity is constrained by ambient vapor pressure, air movement, clothing permeability, and the fraction of skin that is effectively wetted. Thus, two individuals with similar sweat rates can have different cooling outcomes depending on clothing and airflow (Havenith 1999). Thus, physiological coordination is crucial because heat dissipation requires both sweat secretion and the transport of core heat to the body surface. Cutaneous vasodilation increases skin blood flow, raising skin temperature and enabling dry heat exchange when gradients permit. Under substantial heat stress, skin blood flow can reach several l/min, which requires increased cardiac output and places demands on cardiovascular reserve (Périard et al. 2011).

During exercise in the heat, the cardiovascular system faces competing demands: active muscle requires perfusion to sustain work, while skin requires perfusion to dissipate heat. When dehydration reduces plasma volume and venous return, stroke volume can decline and heart rate rises (cardiovascular drift) (Coyle and Gonzalez-Alonso 2001). This can constrain skin blood flow, reduce heat transfer to the shell, and accelerate heat storage. The coupling of thermoregulation to cardiovascular and fluid balance is a central mechanism underpinning both performance impairment and heat illness risk (Crandall and Wilson 2015).

These mechanisms explain why simple ‘air temperature’ thresholds are insufficient. A safe heat environment is one in which required evaporative (Ereq) heat loss is below maximal evaporative (Emax) capacity (Ereq < Emax) for the given activity and clothing. Microclimate factors (humidity, wind, radiation) and behavioral factors (shade, fans, clothing adjustments, pace selection) determine both sides of this inequality.

Comparative work on exercising mammals underscores that there is no single mammalian response to internally generated heat. For a pursuit adapted carnivore such as the African hunting dog (*Lycaon pictus*), body temperature during running is higher and respiratory evaporative heat loss proportionally lower than in domestic dogs, consistent with a water-conserving hyperthermic strategy during endurance locomotion (Taylor et al. 1971). Among ungulates, Thomson’s gazelles store a very large fraction of exercise heat, with rectal temperature increases of more than 4 °C during running (Taylor and Lyman 1972), whereas elands sweat profusely and maintain only small elevations in body temperature; in gazelles, brain temperature rises more slowly than carotid or rectal temperature, consistent with selective brain cooling via the carotid rete (Taylor and Lyman 1972). Classic treadmill studies suggested that cheetahs likewise rely heavily on heat storage and may approach a thermal ceiling during pursuit (Taylor and Rowntree 1973), but field data later showed that free-living cheetahs do not abandon hunts because they overheat, with chase termination occurring at body temperatures near routine daily values and subsequent post-hunt hyperthermia more consistent with stress than with immediate thermal failure (Hetem et al. 2013). Together, these studies indicate that endotherms differ markedly in how they partition exercise heat between storage, evaporation and tissue protection, and they caution against treating any single species, including humans, as a universal model of thermal limitation.

## Microclimate, indices, and why global means mislead

Microclimate refers to the local thermal environment experienced by an organism. For humans this includes air temperature, humidity, more precisely water vapor pressure, wind speed, and radiant heat exchange with sun and surrounding surfaces. Microclimate can vary substantially across distance and time, between sun and shade, between asphalt and grass, between a ventilated street and a sheltered courtyard. Recent commentary (Mitchell et al. 2024) has sharpened this point by arguing that thermoregulatory responses are directly related to responses to the immediate microclimate rather than to regional weather metrics, and that dry heat load is often better captured by globe temperature while evaporative opportunity is better characterized by water vapor pressure than by relative humidity alone.

Because heat exchange depends on multiple variables, several indices have been developed to summarize risk. The wet-bulb globe temperature (WBGT) is widely used in occupational and sporting settings (Brocherie and Millet 2015). WBGT combines natural wet-bulb temperature (humidity + air temperature + wind effects), globe temperature (radiant load), and dry-bulb temperature. Its appeal is operational simplicity, and it underpins many heat policies and standards. However, the WBGT is not a complete physiology model. It does not directly incorporate clothing evaporative resistance, individual metabolic heat production, or interindividual variation in sweating and cardiovascular reserve. For these reasons, WBGT is best viewed as a screening tool rather than a universal predictor of individual tolerance. More detailed approaches, including predicted heat strain models, attempt to estimate T_c_ and sweat loss given activity and clothing, and can be paired with microclimate data for scenario analysis (Lemke and Kjellstrom 2012).

Global mean temperature change is a valuable climate descriptor, but a poor physiological exposure metric. A 1–2 °C increase in global mean does not translate to a uniform shift in local extremes. For instance, heatwaves are episodic, nocturnal minima affect recovery and humidity, wind and radiation modify effective heat load nonlinearly (Jay et al. 2021). The relevant biological question, therefore, is not what global mean temperature is doing in isolation, but how often local microclimates move from compensable to uncompensable conditions for a given organism, activity and clothing state. In other words, climate change threatens human survival not by suddenly invalidating a defended 37 °C T_c_, but by increasing the frequency of local conditions in which required heat loss exceeds achievable heat loss.

## Limits to human heat tolerance: Critical combinations rather than single thresholds

The proposition that there is an upper limit to human heat tolerance has often been framed around wet-bulb temperature (T_w_), which approximates the lowest temperature achievable by evaporative cooling at a given humidity and air temperature. A theoretical heat-balance argument has been used to suggest that sustained exposure becomes unsurvivable for healthy adults at rest near T_w_ ≈ 35 °C, because required evaporative heat loss would exceed what is physiologically possible (Sherwood and Huber 2010). A key development in the subsequent literature has been empirical testing of compensability in climate chambers. These studies typically determine “critical” environmental conditions at which T_c_ begins to rise progressively (uncompensable heat stress) at a defined metabolic rate and air movement (Vecellio et al. 2022). Chamber evidence indicates that critical conditions often occur at T_w_ below 35 °C for typical indoor air movement and light activity, with values around ~ 30–31 °C T_w_ reported under some protocols. This does not contradict the theoretical limit, but it refines it by recognizing that real exposures include finite airflow, clothing, and activity, all of which reduce the effective maximum evaporative potential.

As such, it is more useful to speak of critical combinations rather than a single universal limit. Criticality depends on at least five interacting factors: (i) humidity (vapor pressure), (ii) air temperature, (iii) air movement, (iv) radiant load, and (v) metabolic heat production. Clothing permeability and insulation modulate several of these simultaneously. The same T_w_ can be relatively safe in a breezy, shaded environment and unsafe in still air with substantial radiant heat or impermeable clothing. Radiant heat is especially important in outdoor settings. Direct sun increases heat gain and raises skin temperature, reducing the gradient for dry heat loss and increasing the evaporative requirement. Under these conditions, even moderate T_w_ can become problematic for sustained work or exercise because metabolic heat is superimposed on environmental heat load. Any assessment of heat tolerance that neglects radiation will potentially underestimate the true external burden.

Air movement deserves special emphasis because it can be both protective and harmful depending on thermal gradients. When air temperature is below skin temperature, airflow enhances convection. When air temperature exceeds skin temperature, airflow can increase convective heat gain. However, in many heat-stress scenarios airflow still improves overall heat loss because it accelerates evaporation, provided the air is not near saturation. Thus, the effect of fans or wind cannot be generalized without reference to humidity, air temperature and skin-to-air gradients.

Finally, the physiology of limits is not only thermal but cardiovascular. Compensability requires sustained skin blood flow and sweating. As cardiovascular strain rises (e.g., during exercise or dehydration), skin blood flow may be constrained, raising T_c_ for a given microclimate. Thus, “limits” are better conceptualized as a coupled thermo-cardiovascular boundary rather than a purely thermal boundary (Crandall and Wilson 2015).

### Plasticity: Acclimatization, hydration and behavioral modulation

**Fig. 4:**
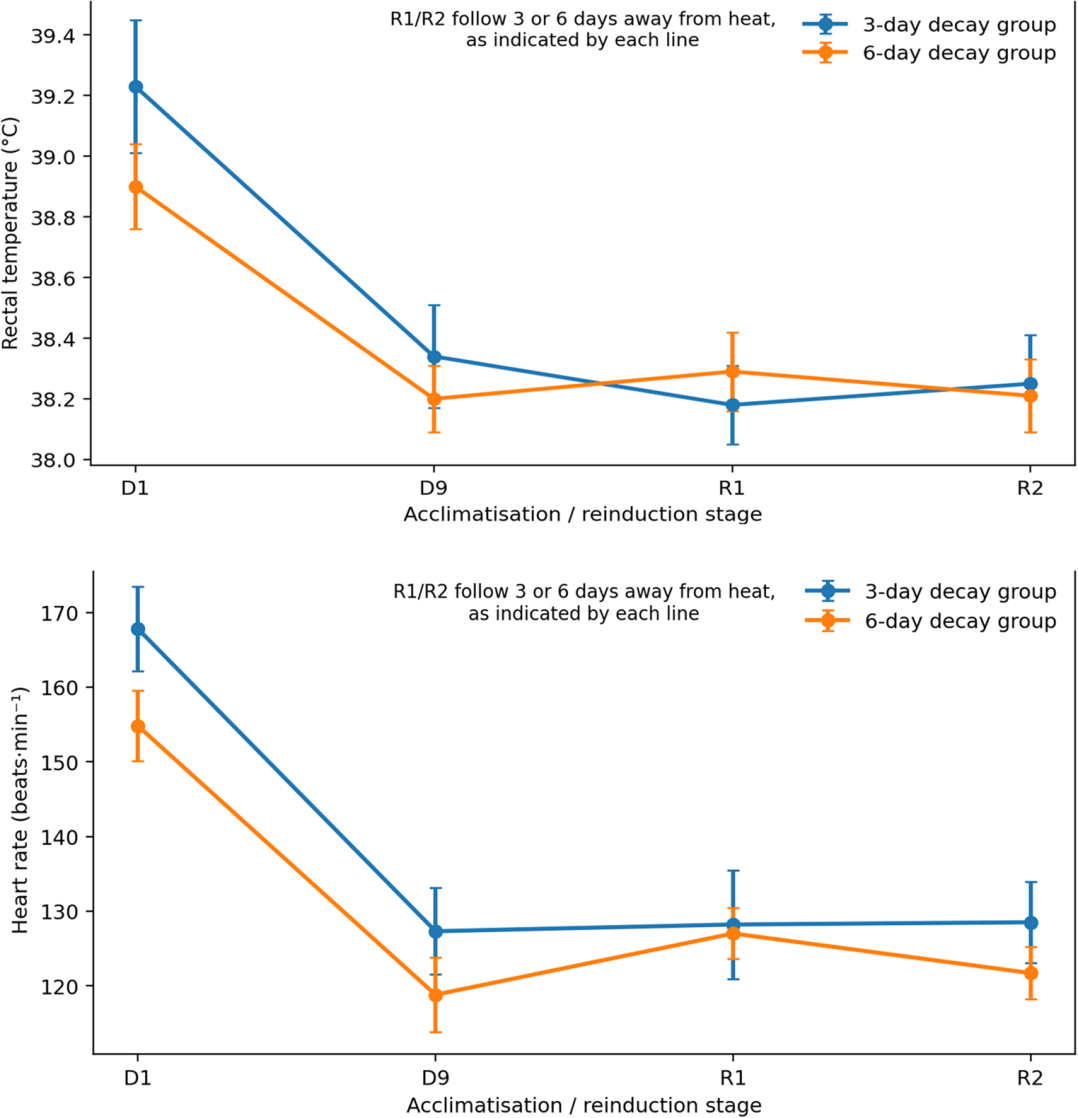
Draft schematic based on Table 1 of Pandolf et al. (1977), showing mean ± SE final rectal temperature (T_re_) and heart rate (HR) after 110 min of walking for the 3-day and 6-day decay groups on the first day of acclimatization (D1), ninth day of acclimatization (D9), and reinduction days 1 and 2 (R1, R2). The figure illustrates reversible physiological plasticity: marked acclimatization from D1 to D9, relatively small decay after several days without heat exposure, and near-restoration of acclimatized responses by the second day of re-exposure. Adapted from subgroup means reported in Table 1. Data redrawn from Pandolf et al. 1977)

Humans exhibit substantial plasticity to repeated heat exposure. Heat acclimatization (field) or acclimation (laboratory) across days to weeks lowers physiological strain at a given workload and microclimate, primarily through cardiovascular and sudomotor adaptations; including plasma volume expansion, reduced heart rate at a given work rate, earlier sweat onset, higher sweat rate, and lower sweat sodium concentration (Taylor 2014). These changes improve the match between Ereq and Emax and delay the onset of uncompensable heat storage. The time course is physiologically important as many adaptations appear within the first week of repeated exposure, with further refinements over ~ 10–14 days with decay occurring with cessation of heat exposure (Pandolf et al. 1977). As shown in Fig. 4, data from the classic acclimatization and reinduction study by Pandolf et al. (1977), shows the time course of changes in T_c_ and heart rate. The data in Fig. 4 indicates that heat acclimatization is not a fixed endpoint but a plastic state that can be retained and rapidly re-induced. In fit men exposed to repeated exercise in severe heat, T_c_ and heart rate fell markedly across nine days of acclimatization, while subsequent losses after 3–6 days away from heat were modest and largely restored within two days of re-exposure. This pattern is useful conceptually because it shows that heat tolerance is dynamic. That is, prior exposure leaves a residual phenotype that can be reactivated rapidly when heat stress returns, particularly when behavioral and hydration opportunities are preserved. Heat acclimatization is, therefore, not a static trait but a reversible phenotype shaped by recent thermal exposure.

Hydration status interacts with acclimatization. Sweating is the dominant cooling pathway, so dehydration reduces cooling capacity both by lowering plasma volume (limiting cardiovascular support for skin blood flow) and by altering sweat responses (Sawka et al. 2012). Even when dehydration does not immediately reduce sweat rate, it can elevate cardiovascular strain and accelerate the rise in T_c_ during prolonged work (Marino et al. 2004). Therefore, availability of water and opportunities to drink are central determinants of whether an otherwise “heat-capable” physiology can be expressed in practice.

Comparative evidence also strengthens the argument that tolerance to heat, and dehydration depends on more than evaporative capacity alone. In hamadryas baboons, 48 h of water deprivation in warm conditions reduced body mass and total body water substantially, yet plasma volume was relatively well conserved, with hematocrit, hemoglobin and blood pressure largely maintained, indicating that circulatory stability itself can be protected under thermal dehydration (Zurovsky et al. 1984). At the same time, work in goats (*capra hircus*) emphasizes that even within a broadly heat-tolerant domestic species, thermoregulatory and metabolic responses vary across breeds, with locally adapted types showing greater tolerance than more heat-sensitive breeds such as Saanen (Lima et al. 2022). These observations reinforce a central comparative point. That resilience to future heat extremes is likely to depend not simply on one effector such as sweating, but on the integrated capacity to preserve circulation, regulate heat exchange, and express appropriate behavioral and metabolic plasticity.

Surface coverings are a further constraint on heat exchange. In humans, clothing insulation is commonly expressed in clo units; even the nude boundary air layer provides about 0.8 clo, and the thermal and evaporative characteristics of a clothing ensemble modify convective, radiative and evaporative heat loss. In empirical manikin studies, vocational clothing systems spanning approximately 1.05–2.58 clo showed substantial differences in evaporative resistance and evaporative cooling efficiency, highlighting how clothing can shift the relation between required and achievable heat loss (Wang et al. 2011; Jacklitsch et al. 2016).

Behavioral thermoregulation is a powerful and often underappreciated form of plasticity. People spontaneously modulate pace, seek shade, change posture, and alter work-rest patterns to maintain tolerable heat strain. In self-paced exercise, reductions in power output can be interpreted as an adaptive behavior that limits heat storage and cardiovascular strain rather than as a simple “failure” of physiology. The same behavioral flexibility can, however, be constrained by external demands (work rate, competition, time pressure), increasing the risk of heat illness.

## Will current human thermoregulatory strategy suffice in a warmer world?

The answer is qualified. Human thermoregulation is not inherently mismatched to a warming climate. That is, eccrine sweating, cutaneous vasodilation, behavioral thermoregulation, and acclimatization provide substantial flexibility, and the continued occupation of hot deserts and humid tropics shows that humans can remain viable across a broad thermal range (Mitchell et al. 2024). But this should not be confused with an unlimited safety margin since these authors argue that relevant exposures are those of the immediate microclimate, in which radiant heat may matter more than air temperature, and in which water vapor pressure and local wind more directly determine evaporative opportunity than headline relative humidity or weather-station values.

**Fig. 5:**
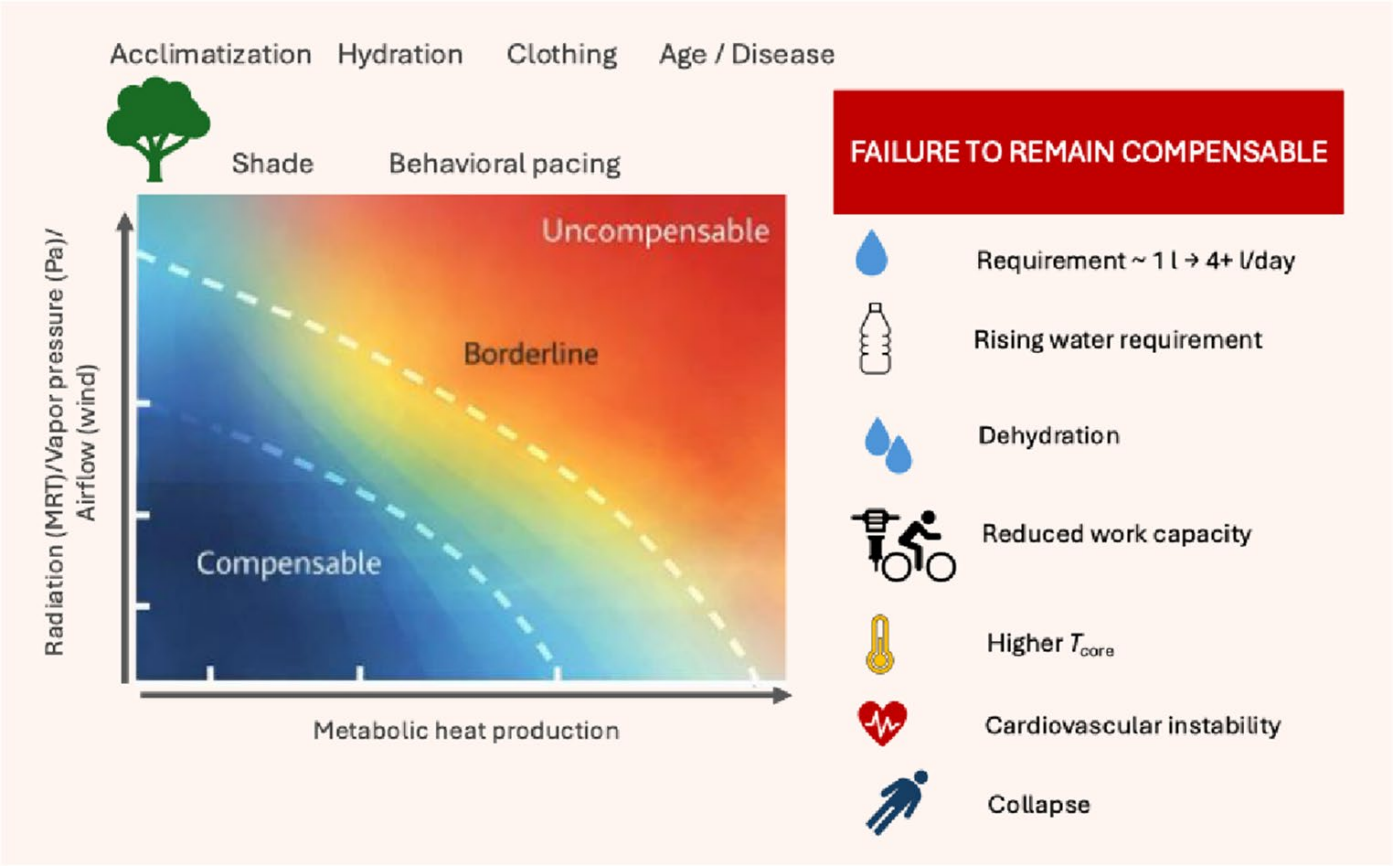
From compensable to uncompensable heat stress: interacting environmental, behavioral and physiological modifiers. Conceptual framework showing the transition from compensable to borderline and uncompensable heat stress as metabolic heat production increases in combination with greater radiant load, vapor pressure and reduced airflow. Acclimatization, shade, hydration, behavioral pacing, clothing or personal protective equipment, and age or disease modify where this transition occurs. Failure to remain compensable is associated with rising water requirements, dehydration, reduced work capacity, higher core temperature, cardiovascular instability and, ultimately, collapse

Recently, this issue has been sharpened from a survival perspective (Tipton and Montgomery 2026). In resting thermoneutral conditions, the minimum average volume to drink is around 1 l day^− 1^, but requirements can increase towards 4 l day^− 1^ and beyond in hot ambulatory conditions. Thus, when that requirement is not met, dehydration raises deep body temperature, reduces work capacity, increases cardiovascular strain and heat illness risk, and in severe circumstances can become fatal within days (Tipton and Montgomery 2026). The implication is that survival in a warmer world depends not only on thermal exposure per se, but on the coupled availability of water, opportunities to reduce radiant and metabolic heat load, and the freedom to modulate exposure by altering behavior (see Fig. 5).

Thus, the most defensible conclusion is that current human thermoregulatory strategy will suffice for many forecast conditions, but only conditionally. It is likely to remain adequate for healthy acclimatized adults when shade, airflow, hydration, reduced activity or paced work, appropriate clothing adjustment and cooler recovery periods remain available. It will be increasingly challenged, and in some circumstances overwhelmed, when combinations of high vapor pressure, low air movement, high radiant load, sleep-disrupting hot nights, clothing or personal protective equipment, and sustained work rate constrain both evaporative and cardiovascular reserve. The experimental task is, therefore, not to ask whether humans can ‘survive climate change’ in the abstract, but to define the microclimatic and resource boundaries at which compensable heat stress becomes uncompensable for different phenotypes, ages and ecological contexts.

## Conclusions

Debates about why mammals defend a T_c_ near ~37°C are best interpreted as reflecting an evolved compromise among biochemical performance, sustained activity, thermal gradient maintenance and host defense rather than a single optimality claim (Tan and Knight 2018) (Schieber and Ayres 2016). The Arrhenius Q_10_ postulate proposed here does not provide a unique explanation, but it does show why a defended temperature in the high-30s °C is biologically plausible under warm microclimatic assumptions. Human thermoregulation is best understood through comparative evolution, microclimate physics and control physiology. The key constraints are not captured by single ambient temperatures or global means, but by the interaction of humidity, radiation, airflow, metabolic heat production, clothing, cardiovascular reserve and access to water.

Framing limits as critical combinations rather than single thresholds clarifies why heat can be tolerable in some apparently extreme environments yet dangerous in others. The thermoregulatory strategy is, therefore, best viewed as conditionally sufficient. It can accommodate many projected exposures when microclimate selection and behavioral adjustment remain possible, but it will fail where local combinations of radiant load, vapor pressure, low wind, sustained activity and restricted access to water or refuge push required heat loss beyond achievable heat loss. For comparative and experimental biology, the central task is to define those boundaries of compensability and the phenotypes most likely to encounter them first.

## Data Availability

Data sharing not applicable to this article as no datasets were generated or analysed during the current development of writing of this manuscript.
